# Electrochemical corrosion behavior of α-titanium alloys in simulated biological environments (comparative study)

**DOI:** 10.1039/d4ra05869k

**Published:** 2024-11-29

**Authors:** Hala Hrir, Omar Ait Layachi, Abderrazzak Boudouma, Abdeslam El Bouari, Abdelaziz Ait Sidimou, Mohssine El Marrakchi, Elmati Khoumri

**Affiliations:** a Laboratory of Physical Chemistry and Biotechnology of Biomolecules and Materials, Hassan II University of Casablanca Faculty of Sciences and Technology Mohammedia 20650 Morocco Halahrir410@gmail.com; b Laboratory of Physical Chemistry, Materials and Catalysis (LCPMC), Faculty of Sciences Ben M’Sik, Hassan II University of Casablanca B.P 7955 Sidi Othmane Casablanca Morocco; c Laboratory of Inorganic Materials for Sustainable Energy Technologies, Mohammed VI Polytechnic University (UM6P) Lot 660-Hay Moulay Rachid Ben Guerir Morocco; d Laboratoire d'Ingénierie des Matériaux pour l'Environnement et les Ressources Naturelles, Université Moulay Ismail de Meknès FST Errachidia BP 509 Boutalamine 52000 Errachidia Marocco

## Abstract

Titanium (Ti) and its alloys are widely utilized in orthopedic and dental applications due to their favorable mechanical properties and biocompatibility. Notably, titanium exhibits excellent corrosion resistance and can form a stable oxide layer, ensuring the longevity and functionality of implants in challenging physiological environments. This study investigates the corrosion behavior of α-Ti alloy in physiological saline solutions, emphasizing the role of key biomolecules found in the human body, including albumin, glycine, and glucose, as well as additional substances such as hydrogen peroxide (H_2_O_2_) and hydroxyapatite (Hap). A comprehensive suite of techniques—namely, open-circuit potential measurements, potentiodynamic polarization, electrochemical impedance spectroscopy (EIS), scanning electron microscopy (SEM), and atomic force microscopy (AFM)—was employed to assess the effects of these biomolecules on corrosion behavior. The findings indicate that, unlike H_2_O_2_ and Hap, the biomolecules studied significantly enhance the corrosion resistance of the α-Ti alloy in simulated physiological environments. H_2_O_2_, due to its strong oxidative properties, accelerates corrosion, while Hap induces ion release that adversely affects the alloy's stability. The observed improvement in corrosion resistance is attributed to the formation of a stable passive layer on the alloy's surface. Notably, this study presents the first long-term electrochemical and immersion tests conducted at 310 K, elucidating the effects of bovine serum albumin (BSA), H_2_O_2_, glycine, glucose, and Hap on the corrosion performance of the α-Ti alloy.

## Abstract 2

This study examines the electrochemical behavior of titanium alloys in simulated physiological solutions using OCP, PPC, EIS, SEM, EDS, and AFM to highlight biomolecule adsorption and TiO_2_ degradation in the presence of H_2_O_2_.

## Graphical abstract

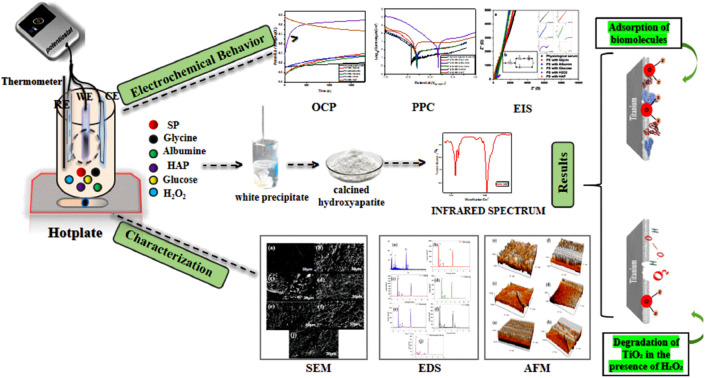

## 1 Introduction

Biomaterials represent a specialized category of materials designed for diagnosing or treating various diseases. Their medical applications include surgical instruments, body tissue replacement and augmentation, controlled drug delivery systems, and tissue engineering scaffolds. From a materials science perspective, biomaterials are classified into five primary groups: polymers, ceramics, metals, plastics, and natural materials.^1^

The corrosion resistance of titanium and titanium alloys is well-documented, primarily due to the formation of a protective oxide film on their surfaces, as confirmed by several studies.^2–4^ Given titanium's extensive use in medical applications,^5^ researchers have thoroughly examined its behavior in environments that simulate the aggressive nature of biological fluids to better understand its corrosion resistance and biocompatibility under physiological conditions. However, the mechanisms by which biomolecules influence the processes of titanium and titanium alloys remain insufficiently understood.^6^

Biomolecules, including proteins and amino acids, can adsorb onto metal surfaces. Additionally, these biomolecules can form films or act as ligands, creating metal complexes on the electrode surface, which may reduce corrosion processes.^7^ This study aims to investigate how albumin, hydrogen peroxide (H_2_O_2_), glycine, glucose, and hydroxyapatite (Hap) affect the corrosion behavior of α-Ti alloy in physiological saline solution (PSS) at 310 K. It also examines how biomolecules, integral to human body compounds, influence corrosion resistance and the dissolution of titanium alloys in saline environments.^5^

The selection of glycine, albumin, glucose, hydrogen peroxide, and hydroxyapatite as media for this study is based on their representation of critical physiological and pathological conditions relevant to orthopedic implants. Glycine and albumin mimic essential biomolecules found in body fluids, while glucose represents metabolic activity. Hydrogen peroxide simulates oxidative stress, and hydroxyapatite reflects the mineral component of bone. By incorporating these diverse elements, this study provides a realistic and comprehensive assessment of the corrosion behavior of the α-titanium alloy in conditions that closely resemble the complex environment surrounding orthopedic implants.

**Fig. 1:**
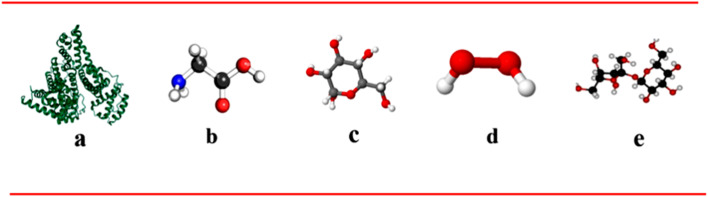
Structures of (a) albumin, (b) glycine, and (c) glucose, (d) H_2_O_2_, (e) hydroxyapatite.

Importantly, this research is the first to conduct a comparative analysis of these media, offering novel insights into how various biomolecules and stressors impact the durability and reliability of titanium implants. This comparative approach yields critical information that can aid in optimizing the design and longevity of implants, ultimately improving clinical outcomes. The structures of albumin, glycine, glucose, H_2_O_2_, and Hap are illustrated in (Fig. 1).

Electrochemical methods are employed to investigate immersions in physiological saline solution (PSS) in order to assess and potentially reverse changes in metal surface deterioration. This deterioration is typically anticipated as a gradual and continuous process following implantation in the body. To evaluate the corrosion behavior of the α-Ti alloy, various electrochemical techniques were utilized, including Electrochemical Impedance Spectroscopy (EIS), Potentiodynamic Polarization Curve (PPC), and Open Circuit Potential (OCP) measurements. These techniques were conducted as a function of immersion period.^1^.

## 2 Experimental protocol

### 2.1 Materials and solution

A plate of α-titanium alloy (composition: Ti 70, Al 5, O 21.6, C 1.5, Na 1, Ca 0.5, Si 0.4) was first fragmented into pieces and then cut into thin plates measuring 5 mm in thickness and 40 mm in length. These fragments were meticulously polished using silicon carbide (SiC) paper to achieve a grain size of 1500, followed by ultrasonic cleaning in a solution of ethanol and bidistilled water.^8^

The next step involved activating the working electrodes in a 1.0 M solution of hydrofluoric acid (HF) for one minute, after which they were carefully rinsed with distilled water.^1^ Electrochemical tests were conducted in a saline solution (0.9% NaCl),^9^ which had an initial pH of 7.01 and was stored in a polyethylene plastic bottle.

To simulate various biological environments, several modifications were made to the saline solution. First, glycine was added at a concentration of 13.32 × 10^−4^ M, which adjusted the pH to approximately 3.^10^ Next, to create a protein-rich environment, albumin was incorporated at a concentration of 40 g L^−1^, resulting in a pH of around 7.4.^11^ Glucose was then added at a concentration of 0.85 g L^−1^ to represent a carbohydrate medium, bringing the pH to approximately 7.2. For simulating an oxidizing environment, hydrogen peroxide (H_2_O_2_) was introduced at a concentration of 35 μM, which lowered the pH to about 6.5.^12^ Finally, hydroxyapatite (Hap), a component found in bone, was added at a concentration of 100 mg L^−1^, adjusting the pH to around 7.6. These modifications to the physiological saline solution provide valuable insights into the inflammatory processes present in the human body.

#### Preparation of Hap

Diammonium Phosphate ((NH_4_)_2_HPO_4_) and calcium nitrate tetrahydrate (Ca(NO_3_)_2_·4H_2_O) were each dissolved separately in water. The resulting solutions were then combined, and the pH was adjusted to a range of 9 to 11 using ammonium hydroxide (NH_4_OH). The mixture was thoroughly stirred, followed by filtration and multiple washes to remove any remaining ammonium ions.

**Fig. 2:**
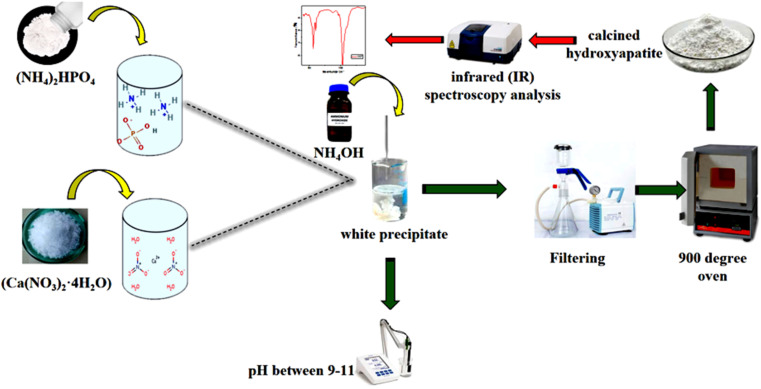
Illustrative diagram of Hap preparation.

The precipitate obtained was subsequently dried and calcined at 900 °C. To confirm the presence of hydroxyapatite (Hap), an infrared spectroscopy (IR) analysis was conducted.^13–15^ An illustrative diagram of the process used to prepare hydroxyapatite is presented in Fig. 2.

### 2.2 Electrochemical tests

**Fig. 3:**
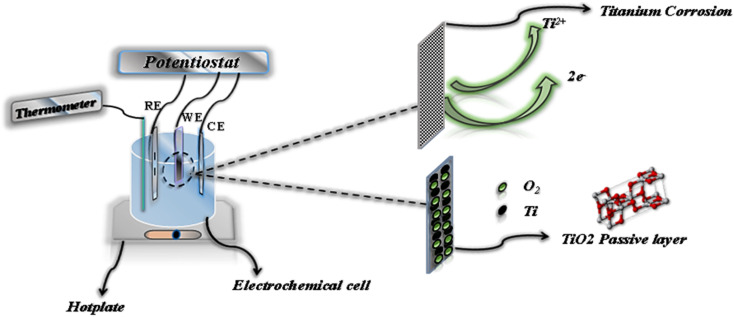
Electrochemical experimental set-up.

Samples for electrochemical testing were encapsulated in epoxy resin, exposing a working surface area of 1 cm^2^. The tests were conducted in a three-electrode electrochemical cell, with the titanium alloy serving as the working electrode, a platinum rod as the counter-electrode, and an Ag/AgCl reference electrode (Fig. 3). The medium used for testing consisted of 100 mL of the solution being studied, which was aerated by exposure to air.

Corrosion measurements were performed using a PalmSens potentiostat. After the samples were fixed, an initial stabilization phase of 30 minutes was allowed, during which the solution was heated to 37 °C to simulate biological conditions. The evolution of the open circuit potential (OCP) was monitored for 3600 seconds at 310 K.^16^

Polarization curves were then plotted within the range of −1 to 1 V (Ag/AgCl) at a sweep rate of 1 mV s^−1^,^8^ from which Tafel lines were derived. Following the OCP measurements, electrochemical impedance spectroscopy (EIS) analysis was conducted. EIS measurements spanned a frequency range from 100 000 Hz to 0.01 Hz, using an alternating sine wave of 10 mV amplitude applied to the electrode at its corrosion potential. The data obtained were used to plot the real (*Z*) and imaginary (*Z*′′) components of impedance, as illustrated in the Nyquist plot.^8,16^

The impedance results were converted into equivalent electrical circuits using PS Trace 5.8 software, enhancing the understanding of how different environments affect titanium. To ensure reproducibility, each experiment was conducted at least twice. The morphology of the sample's surface was examined using a scanning electron microscope (SEM) model Hirox SH-4000M, while an atomic force microscope (AFM) model Nanosurf was employed for further surface analysis.

### 2.3 Immersion tests and surface observation

The surface morphologies of alloy α-Titanium were characterized after a 7 days immersion in various solutions (1). Scanning electron microscopy (SEM) was employed to analyze both the microstructure and surface morphology of the samples. The analysis was performed using a Hirox SH-4000M microscope, equipped with a secondary electron detector (SE), and operated at an electron acceleration voltage of 15 kV to capture SEM images (2). In addition, atomic force microscopy (AFM) was conducted to gather more detailed surface information. This analysis was performed using a Nanosurf model, with an error margin of 10 V (3).

### 2.4 Analysis by infrared spectrometry (FTIR)

Fourier-transform infrared (FTIR) analysis was conducted at room temperature in the mid-IR range (400–4000 cm^−1^). The analysis was performed using a Spectrum Two FT-IR Spectrometer from PerkinElmer (Waltham, MA, USA), equipped with an attenuated total reflectance (ATR) accessory featuring a single reflection diamond crystal.

## 3 Results and discussion

### 3.1 Infrared spectrometry (FTIR) analysis

**Fig. 4:**
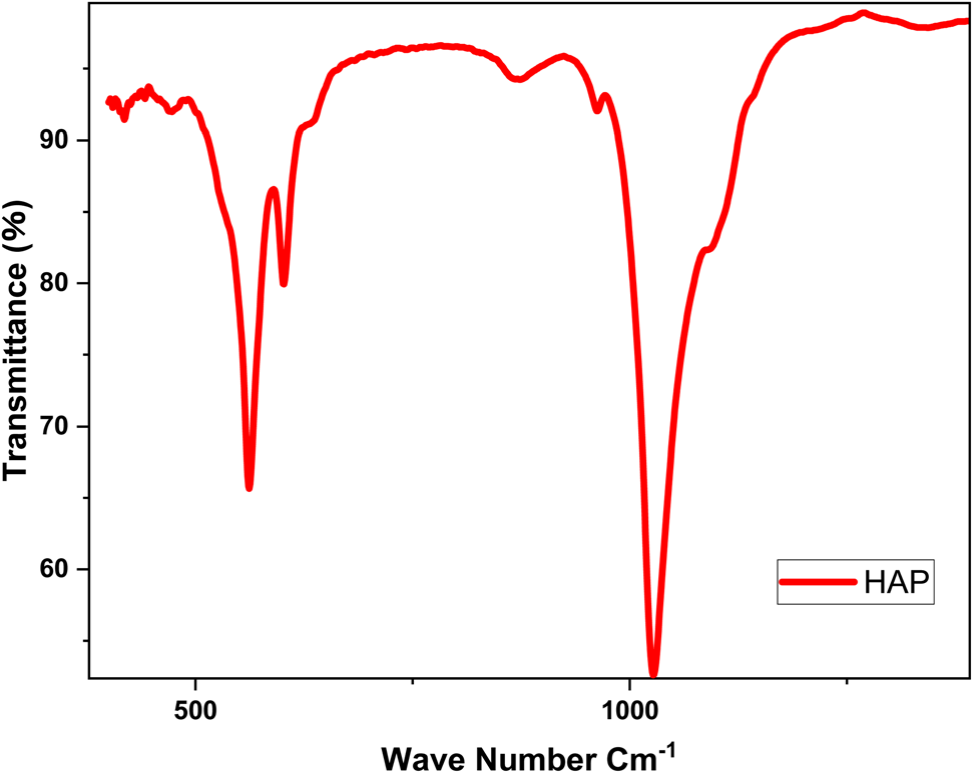
IR spectrum of product calcined at 900 °C.

Infrared (IR) spectroscopy is an analytical technique used to identify and analyze chemical bonds within a material by measuring the absorption of infrared light across different wavelengths. Each chemical bond vibrates at a unique frequency, producing specific absorption peaks in the IR spectrum. ^17^Fig. 4 presents the infrared analysis plot for the functional groups of hydroxyapatite.

The infrared (IR) spectrum of the product prepared and dried at 100 °C, then calcined at 900 °C, displays two distinct peaks at 1000 cm^−1^ and 400 cm^−1^ (Fig. 4). The peak at 1000 cm^−1^ corresponds to the asymmetric deformation vibrations of phosphate groups (PO_4_^3−^), which are essential to the structure of hydroxyapatite (Hap). This peak confirms the presence of phosphate groups, a critical component of Hap. The peak at 400 cm^−1^ is associated with the deformation vibrations of hydroxyl groups (OH^−^) and calcium cations (Ca^2+^) within the Hap crystal lattice. Although less intense, this peak is significant for characterizing the ionic interactions within the hydroxyapatite structure. ^18^ These findings confirm that the prepared sample is hydroxyapatite, supporting its suitability for studying the electrochemical behavior of titanium in biomimetic environments, such as prostheses.

### 3.2 Open circuit potential (OCP)

**Fig. 5:**
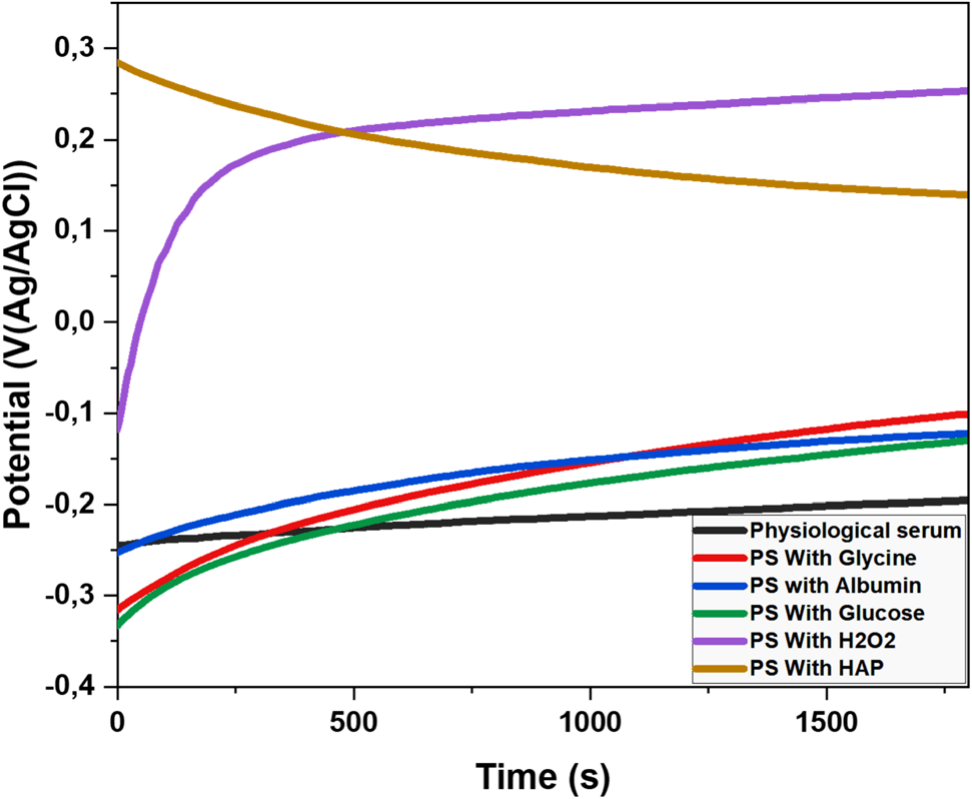
Open-circuit potential curves for Titanium alloy in different solutions: saline solution, amino acid medium (glycine 0.09 g L^−1^), protein medium (Albumin 40 g L^−1^)and glucose (0.85 g L^−1^), H_2_O_2_ (35 μM) hydroxyapatite (0.1 g L^−1^) at 37 °C.

The Open Circuit Potential (OCP) is a key parameter in corrosion studies, representing the electrochemical potential of a material in contact with an electrolyte when no electric current flows across the interface. Measuring the OCP helps evaluate a material's susceptibility to corrosion and provides insights into fundamental corrosion mechanisms. ^19^ This information is essential for developing effective corrosion protection strategies and designing materials resistant to degradation. The OCP of the titanium electrode was monitored in the previously mentioned electrolytes at 37 °C. The results of this observation are presented in Fig. 5.

The corrosion behavior of titanium alloy varies depending on the medium. In physiological saline, the potential shifts from −0.25 V to −0.16 V, indicating slight passivation. These findings are consistent with the results of Ya Liu and Jiuba Wen. ^20^ Glycine strongly promotes passivation, with the potential increasing from −0.32 V to −0.03 V, effectively inhibiting corrosion and significantly enhancing electrochemical stability. Our results align with those of Milan B. Radovanović and Zaklina, 5 who studied titanium in similar solutions containing biomolecules.

Albumin, a protein abundant in blood plasma, causes the potential to rise from −0.25 V to −0.11 V. Its adsorption on the titanium surface forms a physical barrier that reduces access to corrosive ions, though its passivating effect is less pronounced than that of glycine. Similarly, glucose contributes to passivation by increasing the potential from −0.33 V to −0.06 V. It interacts with the titanium alloy surface, modulating the local pH and promoting the formation of a protective oxide layer.

Hydrogen peroxide (H_2_O_2_) demonstrates the strongest passivation effect, with the potential shifting from −0.12 V to 0.28 V. This is due to the rapid and effective formation of a titanium oxide layer. However, the impact of H_2_O_2_ requires careful consideration due to the potential formation of reactive oxygen species (ROS). Our findings regarding H_2_O_2_ are consistent with those of Y. J. Kim and R. A. Oriani. ^21^

The decrease in potential from 0.28 V to 0.15 V in the presence of hydroxyapatite suggests a trend toward corrosion. As the potential drops to 0.15 V, there is a notable loss of stability in physiological serum, reaching a critical point where corrosion becomes significant.

### 3.3 Potentiodynamic polarization curves

The Tafel method is a widely used technique for analyzing the corrosion behavior of metallic materials. It helps determine the kinetics of corrosion reactions by measuring the electrochemical response of a material under varying potential. This method offers valuable insights into corrosion mechanisms, corrosion rates, and supports the development of corrosion-resistant materials. ^22^

**Fig. 6:**
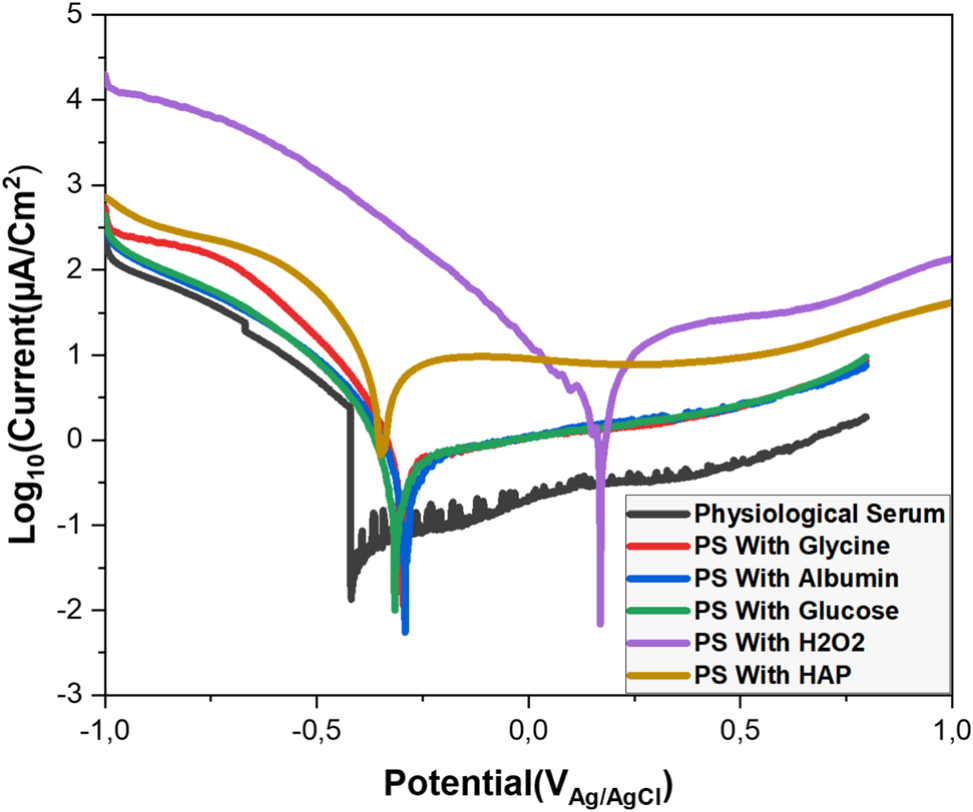
Potentiodynamic polarization curves for Titanium alloy in different media solutions: Saline solution, amino acid medium (glycine 0.09 g L^−1^), protein medium (albumin 40 g L^−1^) and glucose (0.85 g L^−1^), H_2_O_2_ (35 μM) hydroxyapatite (0.1 g L^−1^) at 37 °C.

We conducted Tafel curve analyses on a titanium alloy electrode in various environments (Fig. 6). The analysis provided key parameters related to the corrosion process. The summarized results are presented in Table 1.

**Table 1:** Corrosion parameters of α-titanium in Saline solution, amino acid medium (glycine 0.09 g L^−1^), protein medium (albumin 40 g L^−1^) and glucose (0.85 g L^−1^), H_2_O_2_ (35 μM) hydroxyapatite (0.1 g L^−1^) at 37 °C

	*E*_corr_ (V)	*I*_corr_ (μA cm^−2^)	*β*_a_ (V dec^−1^)	*β*_c_ (V dec^−1^)	*R*_p_ (Ω)	*V*_corr_ (mm per year)
Saline solution	−0.419	0.10	0.150	0.348	29 410	0.001
Glycine	−0.296	3.79	0.120	0.150	69 920	0.033
Albumin	−0.292	6.09	0.200	0.244	74 280	0.053
Glucose	−0.316	0.23	0.180	0.337	92 970	0.002
H_2_O_2_	0.169	94	0.250	0.363	7433	0.791
HAP	−0.35	200	0.220	0.445	4165	1.733

According to the results presented in the table, titanium alloy in saline solution shows moderate corrosion resistance, with low corrosion current, hight polarization resistance and low corrosion rate of 0.001 mm per year (Table 1), indicating the formation of an effective passive layer. These results confirm the observations of ref. 11, which points out that although titanium alloys have good corrosion resistance, exposure to biological fluids such as saline solutions can sometimes result in unexpected behavior, especially under the influence of biomolecules such as albumin and hydrogen peroxide. The effectiveness of the passive layer formed in our study. In the presence of glycine, the corrosion potential becomes more noble at −0.296 V, and the corrosion current decreases, suggesting enhanced passivation. Our findings are consistent with the study by Milan B. Radovanovic, 5 which demonstrated that the presence of biomolecules improves corrosion resistance.

Albumin shows a corrosion potential of −0.292 V and a corrosion current of 6.09 μA cm^−2^, indicating that adsorbed albumin forms a protective barrier on the titanium surface, reducing corrosive damage. These results align with those of Katsuhisa, Masayuki, ^23^ and Nobl F, Magdy A. ^1^

Regarding glucose, the data reveal excellent corrosion resistance due to its favorable interaction with the titanium alloy, promoting the formation of a protective oxide layer.

In contrast, H_2_O_2_ initially shows passivation with a corrosion potential of 0.169 V, but its very high corrosion current and low polarization resistance suggest severe corrosion. This rapid degradation is likely due to the formation of reactive oxygen species (ROS), which accelerate corrosion and compromise the titanium's integrity. Our results are consistent with those of Lidi ^24^ and Nobl F. ^1^

Finally, hydroxyapatite exhibits low polarization resistance of 4165 Ω and a very high corrosion rate, indicating poor stability and high susceptibility to corrosion under these conditions.

### 3.4 Electrochemical impedance spectroscopy

Electrochemical Impedance Spectroscopy (EIS) is a method used to study the electrical response of a system subjected to a sinusoidal perturbation across a range of frequencies. The resistance, as one component of impedance, reflects both the ionic and electrical conductivity of the system. EIS, therefore, allows the analysis of resistance changes with frequency to better understand electrochemical processes and interface reactivity. ^25^

**Fig. 7:**
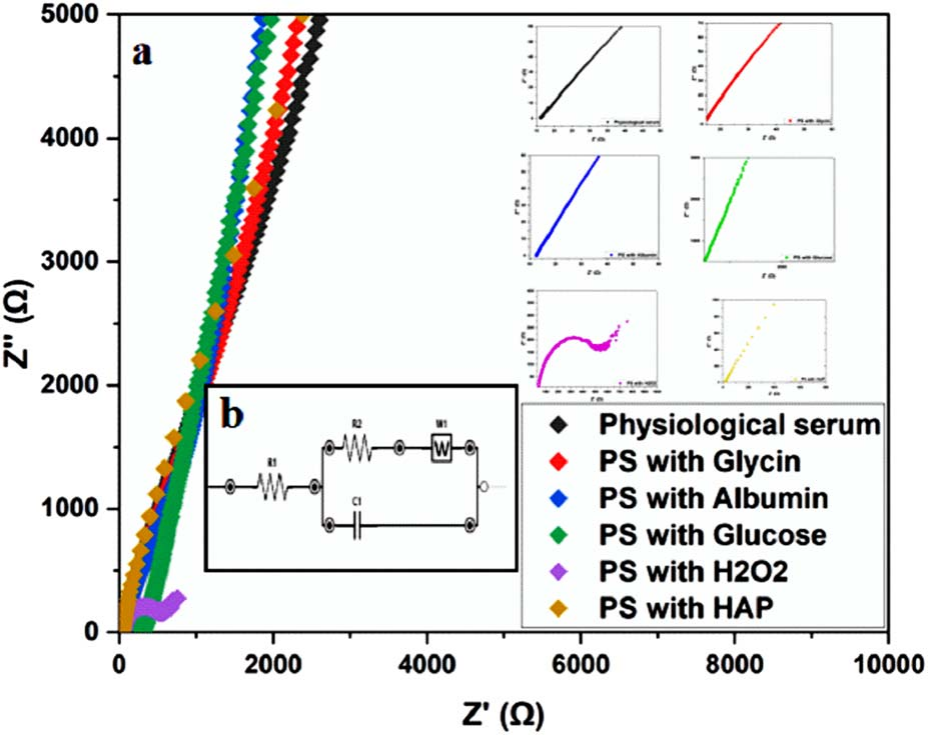
(a) EIS of α-titanium in different solutions: saline solution, amino acid medium (glycin 0.09 g L^−1^), protein medium (albumin 40 g L^−1^) and glucose (0.85 g L^−1^), H_2_O_2_ (35 μM) hydroxyapatite (0.1 g L^−1^) at 37 °C, and EIS a high frequency of the six media, (b) equivalent circuit used to fit the experimental data.

To further examine the effect of different environments on titanium corrosion, EIS measurements were performed in saline solutions containing glycine, albumin, glucose, hydroxyapatite, and H_2_O_2_. The results of these measurements are presented in (Fig. 7) and summarized in (Table 2).

**Table 2:** Adjusted electrochemical parameters for EIS of α-titanium in saline solution, amino acid medium (glycin 0.09 g L^−1^), protein medium (albumin 40 g L^−1^) and glucose (0.85 g L^−1^), H_2_O_2_ (35 μM) hydroxyapatite (0.1 g L^−1^) at 37 °C

	*R*_s_ (Ω)	*C* (μF)	*R*_ct_ (Ω)	*W* (Kσ)
Physiological serum	500	250	3000	5
PS with glycin	150	500	1500	2
PS with albumine	300	5 × 10^5^	3000	2
PS with glucose	200	600	6000	4
PS with H_2_O_2_	150	5000	70	20
PS with HAP	400	500	500	10

The curves obtained by plotting the imaginary part of the impedance against the real part indicate charge transfers at the system interface. For improved visualization, the scales were adjusted to highlight high-frequency regions. These curves are presented in (Fig. 7a). The presence of resistance at both the solution and metal levels can influence the shape of these loops by altering system parameters such as the time constant or frequency response. The analysis of these results leads to the representation of an equivalent circuit, as shown in (Fig. 7b). The electrical parameters derived from this circuit are summarized in (Table 2).

According to the values in (Table 2), the α-Titanium alloy exhibits effective passivation in environments containing physiological serum, glycine, albumin, and glucose. In contrast, the presence of H_2_O_2_ and hydroxyapatite (Hap) in the physiological serum results in significant corrosion of the alloy. The high charge transfer resistance (*R*_ct_) values in passivating environments, as opposed to the low *R*_ct_ values in corrosive environments, support these findings. The equivalent circuit parameters offer valuable insights into the stability of the passivation layer and the effects of various additives on the electrochemical behavior of the alloy.

The observations made in environments containing albumin and H_2_O_2_ align with the study by Nobl F, 1 which also demonstrated the protective role of albumin and the corrosive effects of H_2_O_2_ on metallic alloys.

### 3.5 Evaluation of the surface morphology of titanium alloy

Scanning Electron Microscopy (SEM) is an imaging technique that utilizes an electron beam to examine samples at a microscopic scale. This method provides high resolution and enables the analysis of topography, chemical composition, and material structure. SEM is widely used in various scientific and industrial fields due to its effectiveness in delivering detailed and accurate images of sample surfaces. ^26,27^

**Fig. 8:**
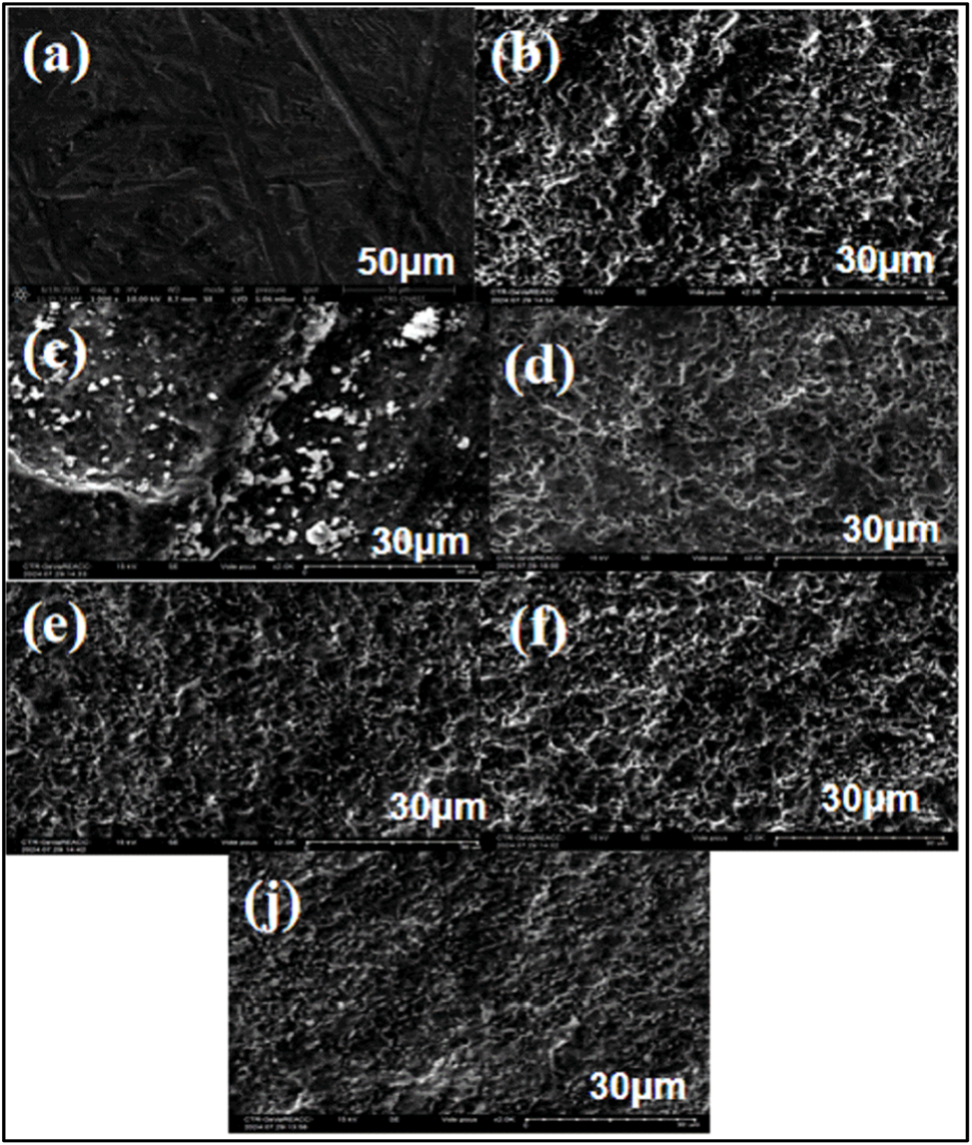
Surface morphology following corrosion of titanium alloy after immersion in solutions for 168 h: (a) titanium alloy before immersion and (b) titanium alloy immersed in SP with glucose, (c) titanium alloy immersed in SP with H_2_O_2_ and (d) titanium alloy immersed in SP with glycine (e) titanium alloy immersed in SP with albumin and (f) titanium alloy immersed in SP with hydroxyapatite, (j) titanium alloy immersed in SP.

In this study, we analyzed the surfaces of a titanium alloy by immersing it in six different environments: saline solution, amino acid medium, protein medium, glucose, H_2_O_2_, and hydroxyapatite. The immersion lasted for 7 days (168 hours). Analyses were conducted before and after immersion, and the resulting images are summarized in (Fig. 8).

The analysis of the images reveals distinct changes in the surface of titanium before and after immersion. Image (a) depicts the initial surface of the titanium, which is smooth and uniform, with no traces of corrosion or deposits. This serves as a reference for evaluating post-immersion alterations.

Image (b) shows the surface of the alloy after immersion in glucose. It displays increased roughness without obvious signs of deep corrosion, suggesting that a protective layer may have formed, inhibiting corrosion.

In Image (c), white particles and clear signs of degradation indicate significant corrosion. This effect is attributed to H_2_O_2_, which promotes titanium oxidation and results in visible surface deterioration.

Image (d) illustrates the surface after immersion in glycine, revealing slight roughness similar to that observed with glucose. The lack of significant corrosion suggests that glycine may inhibit corrosion by forming a protective layer through adsorption.

Image (e) shows that albumin also appears to create a protective layer on the metal. This effect is likely due to the adsorption of amino acids such as cysteine and methionine, which enhance surface passivation.

Image (f) reveals marked roughness and white deposits, indicating significant corrosion. Hydroxyapatite seems to promote corrosion of the titanium alloy, leading to visible surface degradation.

Finally, Image (j) demonstrates that the titanium surface in saline maintains relative stability, with no corrosion detected.

### 3.6 Energy dispersive X-ray spectroscopy (EDS) analysis

Energy Dispersive Spectroscopy (EDS) is an analytical technique utilized to determine the elemental composition of materials. It identifies and quantifies the chemical elements present in a sample by measuring the energy of X-rays emitted by atoms when they are excited by an electron beam. This method is commonly employed to analyze samples on a microscopic scale and provides valuable information on the distribution of chemical elements within materials. ^28^

**Fig. 9:**
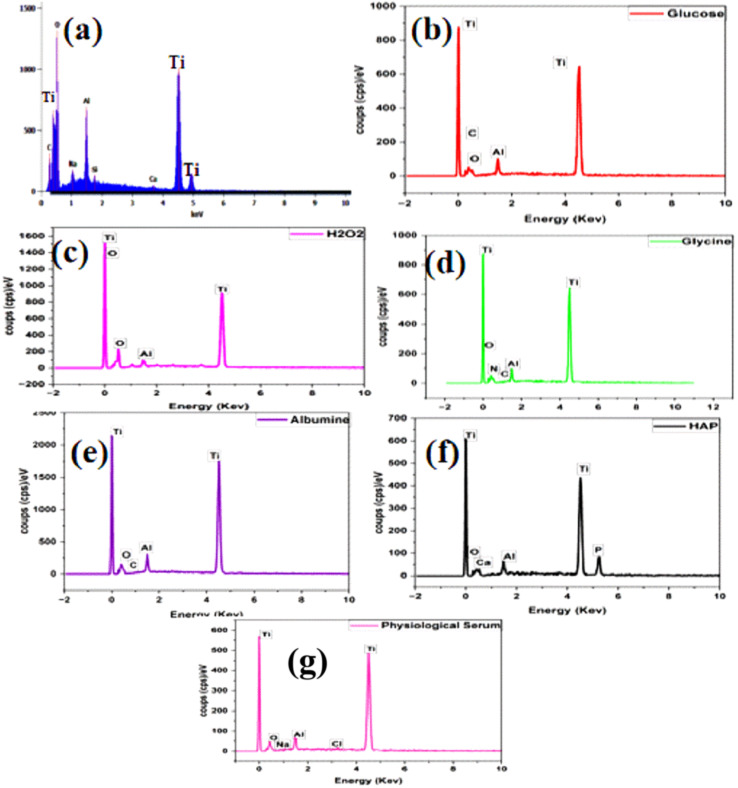
EDS analysis of titanium alloy after immersion in solutions for 168 h: (a) titanium alloy before immersion and (b) titanium alloy immersed in SP with glucose, (c) titanium alloy immersed in SP with H_2_O_2_ and (d) Titanium alloy immersed in SP with glycine (e) titanium alloy immersed in SP with albumin and (f) titanium alloy immersed in SP with hydroxyapatite, (g) titanium alloy immersed in SP.

The elemental composition ratios of samples immersed in different solutions—saline solution, amino acid medium, protein medium, glucose, H_2_O_2_, and hydroxyapatite—after 168 hours are presented in (Table 3) and illustrated in (Fig. 9).

**Table 3:** Elemental composition of titanium alloy

	Ti	Al	O	C	Si	Na	Ca	N	P	Cl
Before immersion	70	5	21.6	1.5	0.4	1	0.5	0	0	0
SP with glucose	77.7	4.88	12.29	5.13	0	0	0	0	0	0
SP with H_2_O_2_	55.95	2.19	41.86	0	0	0	0	0	0	0
SP with glycine	82.79	4.85	8.39	2.71	0	0	0	1.26	0	0
SP with albumine	81.4	4.55	9.86	4.19	0	0	0	0	0	0
SP with Hap	79.15	3.88	15.57	0	0	0	0.7	0	0.7	0
Immersion in SP	89.86	4.6	5.11	0	0	0.28	0	0	0	0.15

The results of the Energy Dispersive Spectroscopy (EDS) analysis reveal significant changes in the elemental composition of the titanium surface before and after immersion in various media. Prior to immersion (Fig. 9a), the initial titanium surface is predominantly composed of titanium, with a notable proportion of oxygen (Table 3). This finding indicates the presence of a natural oxide layer.

In the carbohydrate medium, the EDS analysis shows an increase in titanium and carbon, along with a decrease in oxygen (Fig. 9b). These changes suggest that glucose has adsorbed onto the titanium surface, forming a protective layer that contributes to corrosion inhibition.

Conversely, in the H_2_O_2_ oxidizing medium (Fig. 9c), there is a marked decrease in titanium and a significant increase in oxygen (Table 3). This indicates that H_2_O_2_ caused substantial titanium oxidation, consistent with significant corrosion and without any inhibitory effect.

In the glycine-containing medium (Fig. 9d), the increase in titanium, along with the presence of carbon and nitrogen, suggests that glycine has formed a protective layer on the titanium surface, thereby promoting inhibition of corrosive adsorption.

Similarly, the increased presence of carbon in the albumin medium (Fig. 9e) indicates that albumin also forms a protective layer on the titanium. This layer helps to inhibit corrosion by reducing the availability of oxygen. The elemental ratios observed are comparable to those found with glycine, suggesting similar effectiveness in providing protection.

In the hydroxyapatite-containing medium (Fig. 9f), the presence of calcium and phosphorus, combined with an increase in oxygen (Table 3), indicates that hydroxyapatite may promote corrosion of titanium.

Finally, in the saline-only medium (Fig. 9g), the EDS analysis shows maximum titanium concentration with low oxygen content, indicating surface stability and no noticeable corrosive effect.

### 3.7 Atomic force microscopy (AFM)

Atomic Force Microscopy (AFM) is a surface imaging technique that allows for the visualization and measurement of nanoscale samples. By utilizing an extremely fine probe to “feel” the sample surface, AFM generates detailed three-dimensional images of the surface topography. This technique is particularly effective for studying the mechanical, electrical, and magnetic properties of materials at atomic resolution. ^29–31^

**Fig. 10:**
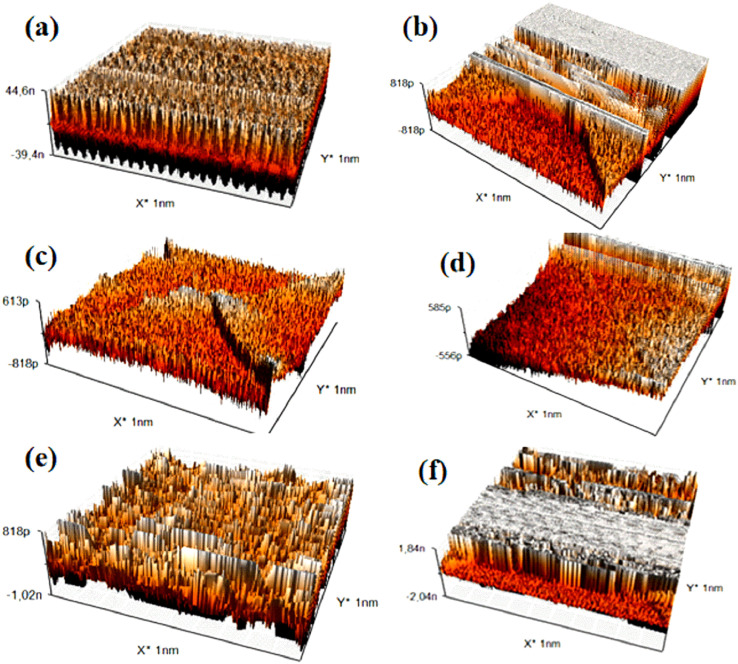
Surface roughness of titanium alloy after immersion in solutions for 168 h: (a) titanium alloy immersed in SP with glucose and (b) titanium alloy immersed in SP with H_2_O_2_, (c) titanium alloy immersed in SP with glycine and (d) titanium alloy immersed in SP with albumin (e) titanium alloy immersed in SP with hydroxyapatite and (f) titanium alloy immersed in SP.

The images and data obtained from the AFM analysis are summarized in (Fig. 10) and (Table 4).

**Table 4:** Surface roughness of titanium alloy (AFM analysis)

	*R*_a_	*R*_q_	*S*_a_	*S*_q_
SP with glycine	0.092 nm	0.114 nm	0.134 nm	0.175 nm
SP with glucose	24.04 nm	26.91 nm	23.63 nm	26.54 nm
SP with albumine	0.101 nm	0.124 nm	0.298 nm	1,.056 nm
Immersion in SP	17.33 nm	20.14 nm	5.47 nm	9.99 nm
SP with HAP	0.176 nm	0.216 nm	0.235 nm	0.311 nm
SP with H_2_O_2_	0.181 nm	0.231 nm	7.26 nm	13.57 nm

The Atomic Force Microscopy (AFM) analysis of titanium alloy samples immersed in different solutions reveals significant variations in surface roughness, indicating differing degrees of protective layer formation or corrosion. The sample immersed in glycine exhibits a relatively rough surface (Fig. 10c), suggesting the development of a protective corrosion-inhibiting layer likely due to the adsorption of glycine molecules. Similarly, samples immersed in glucose (Fig. 10a) and albumin (Fig. 10d) also show increased surface roughness, indicating the formation of protective layers. The albumin layer may be thicker due to the presence of amino acids.

In contrast, samples immersed in hydrogen peroxide (H_2_O_2_) (Fig. 10b) and hydroxyapatite (Fig. 10e) display significantly higher surface roughness, characteristic of considerable corrosion. H_2_O_2_, a strong oxidizing agent, accelerates the degradation of the titanium surface. The saline solution (Fig. 10f), however, shows relatively low surface roughness, which is consistent with the absence of corrosion observed in the electrochemical analysis.

The quantitative roughness results (*R*_a_, *R*_q_, *S*_a_, *S*_q_) presented in Table 4 support these observations. Lower values are recorded for glycine and albumin, indicating enhanced surface protection, while higher values for H_2_O_2_ and hydroxyapatite reflect increased corrosion.

## 4 Conclusion

This study investigated the electrochemical corrosion behavior of α-Ti alloy in a physiological saline solution (PSS) at 310 K, employing techniques such as potentiodynamic polarization curves (PPC), open circuit potential (OCP), electrochemical impedance spectroscopy (EIS), scanning electron microscopy (SEM), energy-dispersive spectroscopy (EDS), and atomic force microscopy (AFM).

The results demonstrate that albumin, glycine, and glucose function as corrosion inhibitors by forming a protective layer that hinders the diffusion of ions and molecules to the titanium surface. This process indicates that the adsorption of these biomolecules on the cathodic active sites effectively reduces cathodic corrosion.

In contrast, the presence of aggressive agents like hydrogen peroxide (H_2_O_2_) and hydroxyapatite (Hap) leads to partial dissolution of TiO_2_, which results in increased corrosion. EIS measurements further confirm the formation of a protective film in the presence of albumin, glycine, and glucose. Conversely, hydrogen peroxide and Hap facilitate both cathodic and anodic reactions, thereby exacerbating corrosion.

Overall, these findings underscore the significant role of biomolecules in enhancing the corrosion resistance of titanium alloys in biomedical applications.

## Data availability

Data will made available on request.

## Conflicts of interest

The authors declare no competing interests.
